# Genome-wide identification of the *restorer-of-fertility-like* (*RFL*) gene family in *Brassica napus* and expression analysis in Shaan2A cytoplasmic male sterility

**DOI:** 10.1186/s12864-020-07163-z

**Published:** 2020-11-04

**Authors:** Luyun Ning, Hao Wang, Dianrong Li, Yonghong Li, Kang Chen, Hongbo Chao, Huaixin Li, Jianjie He, Maoteng Li

**Affiliations:** 1 Department of Biotechnology, College of Life Science and Technology, Huazhong University of Science and Technology, Wuhan, 430074 China; 2 Hybrid Rape Research Center of Shaanxi Province, Shaanxi Rapeseed Branch of National Centre for Oil Crops Genetic Improvement, Yangling, 712100 China; 3 Hubei Collaborative Innovation Center for the Characteristic Resources Exploitation of Dabie Mountains, Huanggang Normal University, Huanggang, 438000 China

**Keywords:** CMS, *Rf*, *RFL*, PPR, RNA-sequencing

## Abstract

**Background:**

Cytoplasmic male sterility (CMS) is very important in hybrid breeding. The *restorer-of-fertility* (*Rf*) nuclear genes rescue the sterile phenotype. Most of the *Rf* genes encode pentatricopeptide repeat (PPR) proteins.

**Results:**

We investigated the restorer-of-fertility-like (*RFL*) gene family in *Brassica napus*. A total of 53 *BnRFL* genes were identified. While most of the *BnRFL* genes were distributed on 10 of the 19 chromosomes, gene clusters were identified on chromosomes A9 and C8. The number of PPR motifs in the BnRFL proteins varied from 2 to 19, and the majority of BnRFL proteins harbored more than 10 PPR motifs. An interaction network analysis was performed to predict the interacting partners of RFL proteins. Tissue-specific expression and RNA-seq analyses between the restorer line KC01 and the sterile line Shaan2A indicated that *BnRFL1*, *BnRFL5*, *BnRFL6*, *BnRFL8*, *BnRFL11*, *BnRFL13* and *BnRFL42* located in gene clusters on chromosomes A9 and C8 were highly expressed in KC01.

**Conclusions:**

In the present study, identification and gene expression analysis of *RFL* gene family in the CMS system were conducted, and seven *BnRFL* genes were identified as candidates for the restorer genes in Shaan2A CMS. Taken together, this method might provide new insight into the study of *Rf* genes in other CMS systems.

**Supplementary Information:**

Supplementary information accompanies this paper at 10.1186/s12864-020-07163-z.

## Background

The male sterile line was widely used in hybrid breeding, which mainly included chemical induced male sterility (CIMS), genic male sterility (GMS) and cytoplasmic male sterility (CMS) [1, 2]. In CMS, traits are maternally inherited, primarily due to the rearrangement of mitochondrial DNA and inability to generate normal pollen [3]. The restorer-of-fertility (*Rf*) nuclear genes have been used to rescue the damage induced by mitochondrial DNA rearrangements. In *Brassica napus*, there are four major CMS systems which have been commonly used in rapeseed production: *pol* CMS [4], *nap* CMS [5], *Ogu* CMS [6], and Shaan2A CMS [7]. Shaan2A CMS and *pol* CMS are the most widely used CMS systems in *B. napus* [8]. What’s more, in Shaan2A CMS system, the cytoplasm type of its restorer line KC01 belongs to *pol* CMS type [9].

The first *Rf* gene encoding a putative aldehyde dehydrogenase was cloned in the T-CMS of maize (*Zea mays*); the encoded protein either performs acetaldehyde detoxification or interacts with the male sterile mitochondrial proteins [10]. To date, many other *Rf* genes have been identified in different CMS systems. Most of these *Rf* genes encode pentatricopeptide repeat (PPR) proteins. Examples of such *Rf* genes include *Rf-PPR592* in petunia (*Petunia hybrida*) [11], *Rfo* [12] and *orf687* [13] in radish (*Raphanus sativus*), *Rf4* [14], *Rf5* [15] and *Rf6* [16] in rice (*Oryza sativa*) and *Rfp* [17] and *Rfn* [18] in rapeseed. The PPR proteins were first identified as tandem repeats of degenerate 35-amino-acid motifs (PPR motifs) in *Arabidopsis thaliana* [19] and were classified into PLS and P subfamilies, according to the PPR motif structure [20]. The *PPR* gene family is a large family comprising 441 members in *Arabidopsis* [21], 491 members in rice [22] and 626 members in poplar (*Populus alba*) [23]. Except for the PPR13 in sorghum (*Sorghum bicolor*), most of the RF-related PPR proteins belong to the P subfamily and lack the catalytic sites for RNA editing or binding [15]. Two partner proteins of the RF-related PPR proteins have been reported, including GRP162, which associates with RF5 [15], and hexokinase 6 (HXK6), which functions together with RF6 [16] to rescue CMS in rice.

To date, all reported *Rf* genes have been identified via genetic mapping, which is a time-consuming method and takes several years to narrow down the genomic region of interest. However, the concept of restorer-of-fertility-like (*RFL*) gene was put forward in 2011, and 212 *RFL* genes were identified based on BLAST searches using the *Rf-PPR592* and *Rf5* sequences against the genomes of 13 different dicot and monocot species, including *Arabidopsis*, soybean (*Glycine max*) and sorghum [24]. AtRFL2, together with RNase P, regulates the processing of mitochondrial *orf291* RNA [25]. AtRFL4 is needed for processing the 5′-end of *nad4* mRNA in mitochondria [26]. AtRFL9, also known as RNA PROCESSING FACTOR 4 (RPF4), participates in the generation of extra 5′ termini of ccmB transcripts in *Arabidopsis* [27]. These results enhanced our understanding of mitochondrial RNA processing in plants and provided novel insights into the function of RFL proteins.

In the present study, we performed BLAST searches using the sequences of *Rf-PPR592* and *AtRFLs* against the genome of rapeseed and identified 53 *BnRFL* genes. Based on these 53 *BnRFL* genes, candidate *Rf* genes were analyzed in the Shaan2A CMS system by RNA-seq and tissue-specific expression analyses. Our data provide a strong foundation for the study of *Rf* genes in other CMS systems.

## Results

### Identification of *BnRFL* genes

A total of 53 *BnRFLs* were identified in this study, based on the homology with the *RFL* genes in *Arabidopsis* (*AtRFL1–26*) and petunia (*Rf-PPR592*) (Table 1). First, sequences of all 26 *AtRFLs* were searched in the database one at a time. Of the 26 *AtRFL* genes, nine showed no homologs in *B. napus*, including *AtRFL5*, *AtRFL6*, *AtRFL9*, *AtRFL10*, *AtRFL14*, *AtRFL15*, *AtRFL16*, *AtRFL25* and *AtRFL26*. Then, using *Rf-PPR592* as a reference [24], 26 *BnRFLs* were identified (E-value <1e^− 100^). Taken together, there should be a total of 53 *BnRFLs* genes in *B. napus*. We also identified two known restorer genes, *BnRFL6* (*Rfn*) and *BnRFL13* (*Rfp*) (previously identified in the *nap* and *pol* CMS systems) and four candidate restorer genes (*BnRFL2*, *BnRFL10*, *BnRFL11* and *BnRFL42*; previously identified in *B. napus* by fine genetic mapping) [31].

**Table 1:** Summary of the chromosomal location of *BnRFL* genes and characteristics and subcellular localization of the encoded proteins

*A. thaliana* gene	*A. thaliana* ID	*B. napus* gene	Chr.	Gene position		PPR number	Protein Length (AA)	pI [28]	Molecular weight (Da) [28]	GRAVY [28]	Subcellular location	
				Start	End						Pprowler [29]	TargetP [30]
AtRFL1	At1G06580	BnRFL1	C8	45,301,508	45,303,299	11	497	8.55	56,608.61	0.183	M^b^	M
AtRFL2	At1G12300	BnRFL2	A9	42,781,901	42,784,196	15	637	5.97	70,948.87	0.026	M	M
		BnRFL3	A9	42,776,628	42,778,959	15	647	6.31	71,868.6	0.038	M	M
		BnRFL4	C8	42,874,876	42,877,570	14	640	5.61	71,088.91	0.032	M	M
		BnRFL5	C8	42,856,820	42,859,076	15	626	6.2	69,734.49	0.01	M	M
		BnRFL6	A9	42,851,654	42,853,921	15	629	8.33	69,640.9	0.16	M	M
		BnRFL7	A1	25,159,038	25,161,294	15	626	6.16	70,426.2	0.001	M	S^c^
		BnRFL8	C8	42,947,165	42,949,404	15	621	7.34	68,638.38	0.116	M	M
AtRFL3	At1G12620	BnRFL9	A8	25,819,298	25,821,504	14	612	6.65	68,324.47	−0.043	M	M
		BnRFL10	Un^a^	20,135	22,471	15	648	8.21	72,194.66	0.043	**—**^**d**^	C^e^
		BnRFL11	A9	42,353,345	42,355,681	15	648	8.2	72,022.51	0.067	**–**	C
		BnRFL12	C8	17,918,175	17,920,367	15	608	7.37	67,610.72	−0.023	M	M
		BnRFL13	A9	42,707,709	42,710,051	15	650	8.41	73,339.39	−0.054	M	M
		BnRFL14	C8	17,950,498	17,951,759	8	323	4.67	35,505.17	0.06	**–**	**–**
		BnRFL15	C8	42,022,999	42,023,776	5	215	5.29	23,581.51	0.067	M	S
AtRFL4	At1G12700	BnRFL16	C8	42,872,137	42,874,315	14	604	5.47	67,048.93	0.02	M	M
		BnRFL17	A9	42,773,730	42,775,903	14	603	6.57	66,969.91	−0.018	M	M
AtRFL7	At1G62680	BnRFL18	Un	30,312	32,846	12	538	8.45	60,152.19	0.016	M	C
		BnRFL19	A9	7,486,280	7,488,209	12	535	8.56	59,967.03	0.012	M	M
		BnRFL20	A9	9,401,981	9,403,866	12	523	8.33	58,558.11	−0.002	M	M
		BnRFL21	A9	9,446,842	9,448,591	11	440	4.81	48,891.56	0.127	**–**	M
AtRFL8	At1G62720	BnRFL22	A9	7,519,673	7,521,422	13	485	8.39	55,092.25	−0.012	M	C
AtRFL11	At1G62930	BnRFL23	C1	45,473,258	45,480,422	15	487	6.36	55,189.36	−0.013	S	S
		BnRFL24	A9	9,204,272	9,204,884	4	178	5.29	20,006.32	0.001	**–**	**–**
AtRFL12	At1G63070	BnRFL25	A9	9,203,551	9,204,328	4	188	5.47	21,000.35	−0.047	**–**	**–**
AtRFL13	At1G63080	BnRFL26	C4	53,751,551	53,751,949	2	107	6.26	11,658.37	−0.133	**–**	**–**
		BnRFL27	C4	53,761,784	53,762,107	2	107	6.26	11,658.37	−0.133	**–**	**–**
AtRFL17	At1G63400	BnRFL28	C1	24,931,745	24,934,613	19	796	6.17	89,097.00	−0.119	M	C
		BnRFL29	A1	15,383,421	15,386,289	18	796	6.8	88,897.86	−0.12	M	C
AtRFL18	At1G64100	BnRFL30	A9	9,036,004	9,038,611	17	681	5.94	76,309.56	0.017	M	M
		BnRFL31	A9	9,033,346	9,035,550	17	681	5.94	76,309.56	0.017	M	M
		BnRFL32	C1	45,499,113	45,501,687	17	681	6.24	76,106.47	0.022	M	M
AtRFL19	At1G64580	BnRFL33	A9	8,665,312	8,667,139	12	507	9.1	57,063.97	−0.01	M	M
		BnRFL34	C5	40,329,839	40,331,444	10	445	9.16	50,074.12	0.009	**–**	**–**
		BnRFL35	Un	47,833	52,673	12	491	9.05	55,206.81	0.062	M	M
		BnRFL36	A9	9,201,254	9,203,025	12	491	9.04	55,221.77	0.036	M	M
		BnRFL37	Un	21,883	23,773	10	445	9.16	50,074.12	0.009	**–**	**–**
AtRFL20	At3G16710	BnRFL38	A1	29,342,555	29,344,457	12	506	8.64	57,356.77	0.032	M	M
		BnRFL39	Un	67,690	69,826	10	453	8.94	51,204.74	0.017	M	C
AtRFL21	At3G22470	BnRFL40	A8	25,799,162	25,801,437	13	631	6.99	70,631.48	0.008	M	M
		BnRFL41	C8	42,220,823	42,223,242	15	671	8.44	75,390.4	−0.045	M	**–**
		BnRFL42	A9	42,760,108	42,762,458	15	652	6.73	73,148.36	−0.005	M	M
		BnRFL43	C1	14,072,487	14,074,026	11	423	5.4	46,851.41	−0.088	**–**	–
		BnRFL44	A8	25,805,814	25,806,641	5	229	4.63	25,248.08	−0.064	**–**	**–**
		BnRFL45	C8	17,950,054	17,950,652	4	165	8.12	18,161.4	0.335	S	S
AtRFL22	At4G26800	BnRFL46	A1	11,953,788	11,955,598	10	502	8.86	56,499.9	−0.017	M	M
		BnRFL47	C1	20,149,199	20,151,006	11	501	8.94	56,441.83	0	M	M
AtRFL23	At5G16640	BnRFL48	Un	18,562	20,747	12	501	8.46	56,334.67	0.011	M	C
		BnRFL49	A10	15,694,475	15,696,271	11	498	8.73	56,128.71	0.018	M	C
AtRFL24	At5G41170	BnRFL50	Un	83,037	85,098	11	481	8.76	54,110.04	0.023	M	M
		BnRFL51	A4	11,026,724	11,028,448	11	478	8.84	53,924	0.064	M	M
		BnRFL52	A9	27,526,100	27,529,023	16	811	7.06	92,429.64	−0.364	M	M
		BnRFL53	C7	55,839,984	55,843,142	14	821	7.89	93,225.71	−0.308	M	M
Rf-PPR592 [11]						14	592	7.81	67,340.37	−0.071	M	M
Rf4 [14]						18	782	6.56	86,282.74	−0.037	M	M
Rf5 [15]						17	791	6.1	87,614.43	−0.013	M	M
Rf6 [16]						15	798	8.4	88,617.48	−0.096	M	M
Rfo [12]						17	687	4.96	76,500.42	0.022	M	M

a Unplaced Scaffold

b Mitochondrion

c Secretory pathway

d Any other location

e Chloroplast

The number of PPR motifs in the BnRFL proteins varied from 2 to 19, although most of the BnRFL proteins contained at least 10 PPR motifs and the average number of PPR motifs was 12 (Table 1). Approximately one-fifth of the BnRFLs showed relatively low pI (< 6), whereas nearly half of the BnRFLs showed relatively high pI (> 8). The molecular weight of these RFL proteins ranged from 11.7–92.4 kDa. Additionally, the GRAVY value of nearly two-fifth BnRFLs and most of the selected *Rf* genes was less than 0, indicating that these RFL proteins were hydrophilic. Most of the BnRFLs were predicted to localize to the mitochondria, which is consistent with the subcellular localization of RF proteins (Table 1).

### Chromosomal location and structural analysis of *BnRFL* genes

**Fig. 1:**
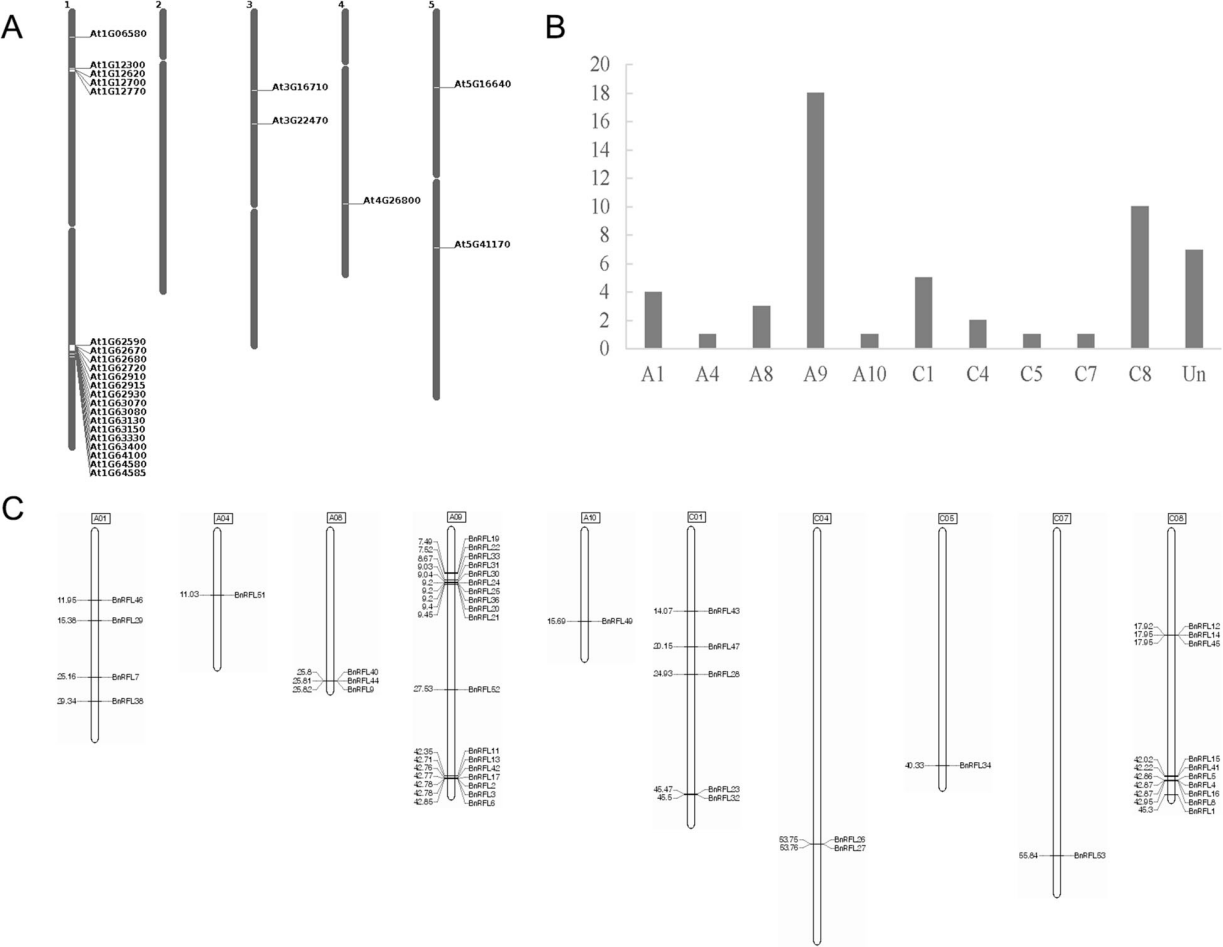
Distribution of the *AtRFL* and *BnRFL* genes on chromosomes. **a** Chromosomal distribution of *AtRFLs*. **b** Number of *BnRFLs* on different chromosomes. **c** Chromosomal distribution of *BnRFLs*

First, we downloaded the chromosomal distribution of *AtRFLs* from The Arabidopsis Information Resource (TAIR) (Fig. 1a). All 26 *AtRFLs* were located in a cluster on chromosome 1. Of the 53 *BnRFLs* identified in this study, 46 *BnRFLs* were distributed unevenly on 10 of the 19 chromosomes, and 18 and 10 *BnRFLs* formed highly dense clusters on chromosomes A9 and C8, respectively. The remaining seven *BnRFLs* were located on the unmapped scaffold (Fig. 1b and c).

Next, we determined the exon-intron structure of the *BnRFL* genes and a few known restorer genes (Additional file 1). Most of the *BnRFLs* were intron-less, similar to the restorer genes, such as *Rf4*, *Rf5* and *Rf6*, in rice CMS line. Of the 53 *BnRFL* genes, 10 contained a single intron, similar to the *Rf* genes, *Rfk1*, *Rfob* and *orf687*, in radish. Notably, the intron in *BnRFL23* was more than 4 kb in length, unlike other *BnRFLs*.

**Fig. 2:**
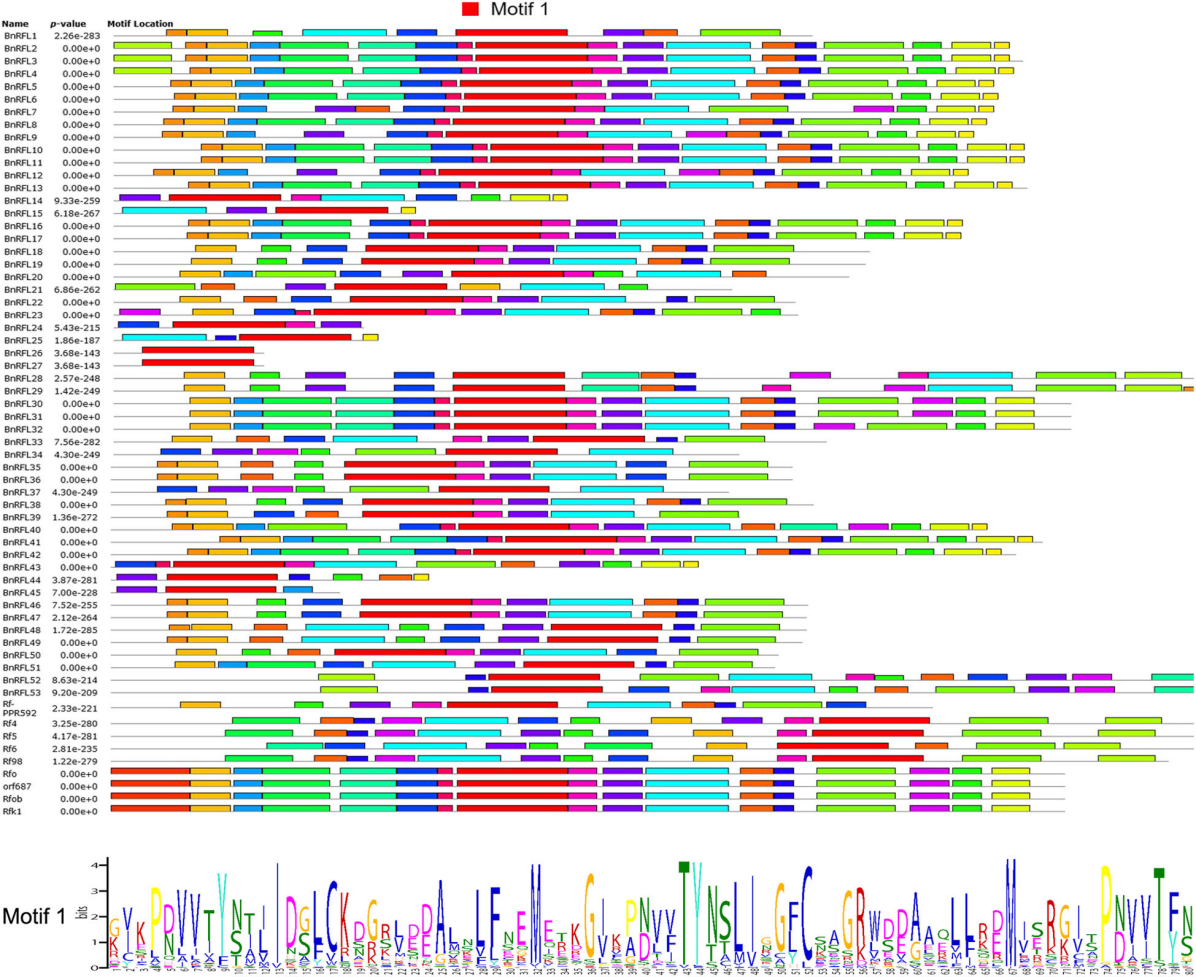
Distribution of 20 motifs identified in BnRFL proteins, and sequence of the conserved motif 1

Because PPR proteins generally contain tandem repeats of PPR motifs, we searched for the PPR motifs in the BnRFL proteins and a few known restorer proteins (Additional file 2). To investigate whether BnRFLs contained additional motifs, 53 BnRFLs and 9 known restorer proteins from other species were submitted to MEME. The results showed 20 motifs in the BnRFL proteins (Fig. 2). Interestingly, all of the identified RFL proteins contained motif 1, which contained 80 amino acids.

### Phylogenetic and Syntenic analysis

**Fig. 3:**
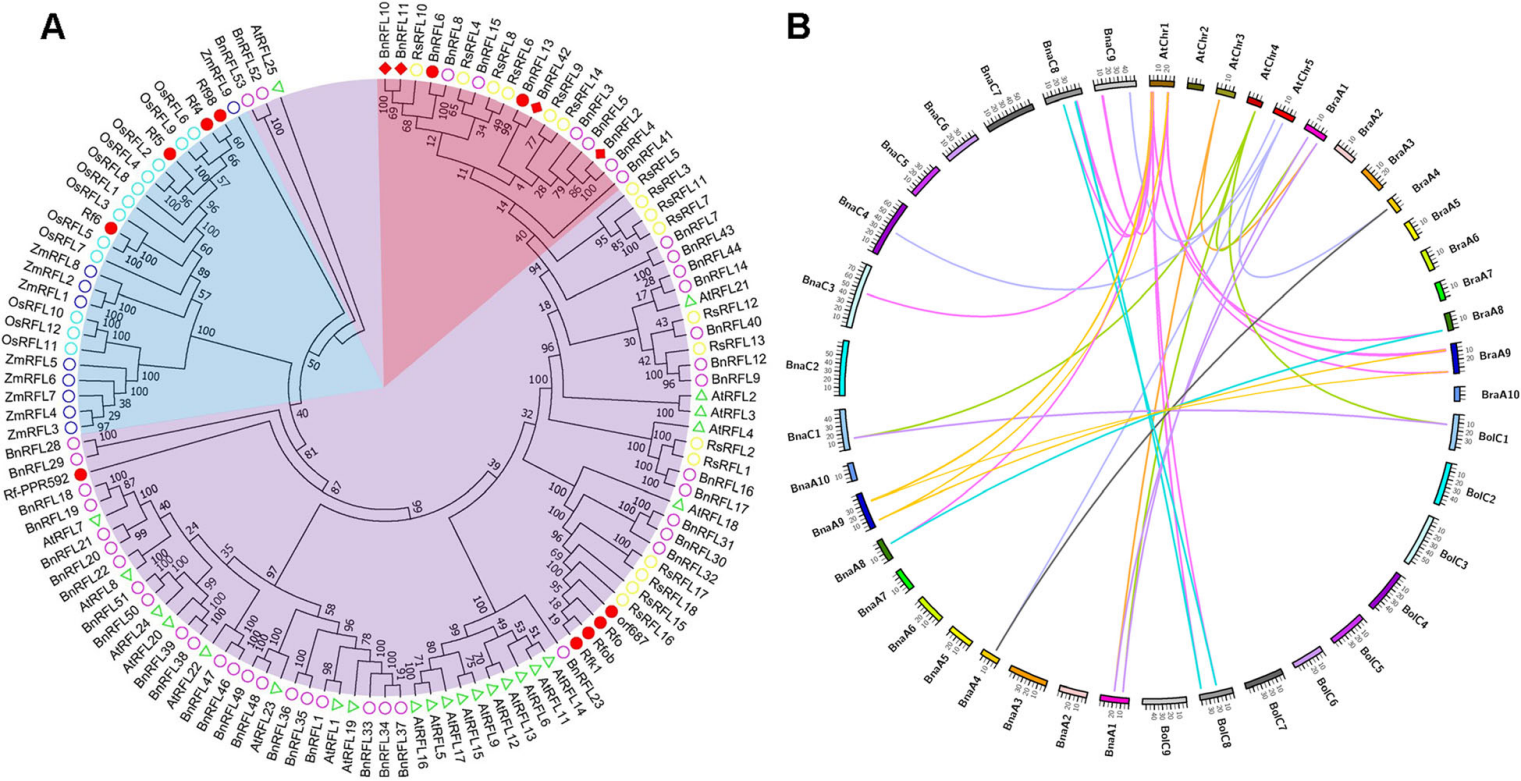
Phylogenetic and syntenic analysis of *RFL* genes. **a** Phylogenetic analysis of RFLs in rapeseed (*Brassica napus*), *Arabidopsis thaliana*, rice (*Oryza sativa*), maize (*Zea mays*) and radish (*Raphanus sativus*). Monocot RFLs are indicated in light blue. Dicot RFLs are indicated in light purple. RFLs in the cluster of known RF proteins are indicated in pink. Red solid circles, known RF proteins; red diamonds, candidate RF proteins in a previous study. Yellow, purple, light blue and dark blue circles and green triangles represent the *RFL* genes in *R. sativus*, *B. napus, O. sativa, Z. mays* and *A. thaliana*, respectively. Numbers at nodes (range 0–100) indicate the reliability of the corresponding branch; higher the number, higher the reliability of the branch. **b** Synteny analysis of the *RFL* genes in *A. thaliana*, *B. napus*, *B. oleracea* and *B. rapa*. AtChr1–5, *A. thaliana* chromosomes 1–5; BraA1-A10, *B. rapa* chromosomes 1–10; BolC1-C9, *B. oleracea* chromosomes 1–9; BnaA1-A10, *B. napus* chromosomes A1-A10; BnaC1-C9, *B. napus* chromosomes C1-C9

To identify the homologs of BnRFLs in different monocot and dicot species, multiple sequence alignments were performed and sequence similarity was determined. The rice RF5 protein was used for BLAST searches against the rice and maize genomes, and Rf-PPR592 was used for BLAST searches against the radish genome. An additional 16 OsRFLs (including 4 reported restorer genes), 9 ZmRFLs, and 22 RsRFLs (including 4 known restorer genes) were identified (E-value <1e^− 100^). Phylogenetic analysis revealed that RFLs mainly formed two separate clusters, and RFLs in monocot and dicot species were clustered together, respectively (Fig. 3a). Additionally, two reported restorer genes (*BnRFL6* and *BnRFL13*) and four candidate restorer genes (*BnRFL2*, *BnRFL10*, *BnRFL11* and *BnRFL42*) clustered together. Six additional *BnRFLs* (*BnRFL3*, *BnRFL4*, *BnRFL5*, *BnRFL8*, *BnRFL15* and *BnRFL41*) clustered together with the reported and candidate restorer genes. These 12 *BnRFLs* have been deeply investigated in the following study.

Next, we examined the synteny of *BnRFLs* with their homologs in *Arabidopsis*, *B. rapa* and *B. oleracea* (Fig. 3b). The results showed syntenic relationships between *AtRFL* genes on chromosome 1 and *RFL* genes on chromosomes BraA8, BraA9, BolC8, BnaA8, BnaA9, BnaC3, BnaC8 and BnaC9. The *AtRFL* gene on chromosome 3 showed synteny with *RFL* genes on BraA1 and BnaA1. The *AtRFL* genes on chromosome 4 showed synteny with *RFL* genes on BraA1, BolC1, BnaA1 and BnaC1, and the *AtRFL* genes on chromosome 5 showed synteny with *RFL* genes on BraA4, BnaA4, BnaC4 and BnaC9.

The Ks and Ka values indicate the evolutionary pressure on species. A Ka/Ks ratio < 1indicates functional constraint, whereas Ka/Ks ratio > 1 indicates positive selection [32]. To explore the selection pressure on *BnRFLs*, we calculated the Ka/Ks ratios (Additional file 3). All *BnRFL* genes showed a Ka/Ks ratio of 0.1–0.7. The KaKs ratio of most of the *BnRFLs* was relatively low (< 0.4). However, *BnRFL46* and *BnRFL47* showed relatively high Ka/Ks ratios (> 0.6).

### Interaction analysis of AtRFL proteins

**Fig. 4:**
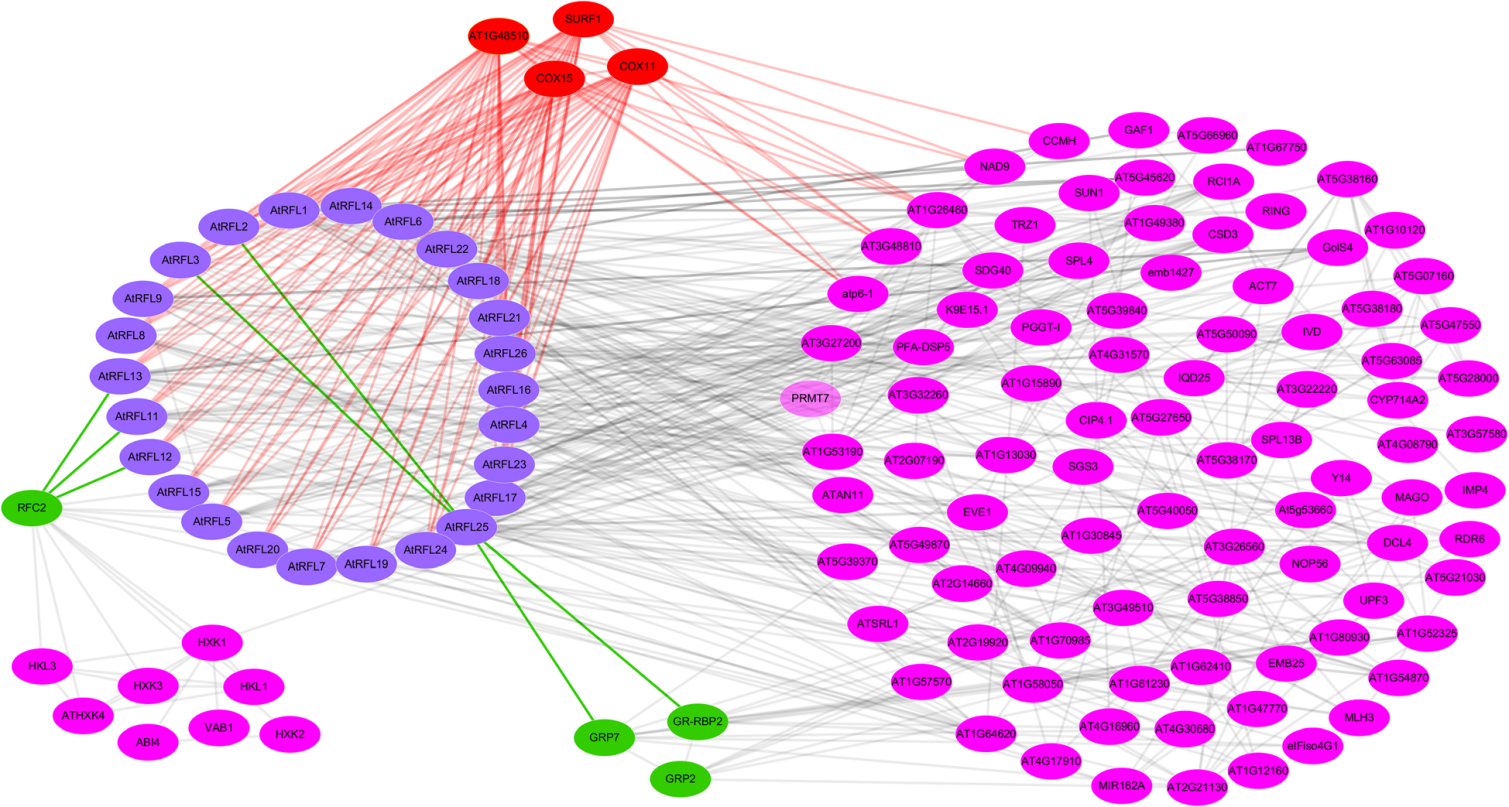
Interaction analysis of AtRFLs. Purple nodes represent AtRFLs. Proteins interacting with AtRFLs, shown in other colors, were searched in the STRING database

Most of the RFL proteins belong to the P subfamily and need to interact with other proteins to perform RNA processing [15]. To predict the interacting partners of RFL proteins (no *B. napus* information in STRING database), an interaction network for AtRFLs were constructed based on STRING 10.5 and Cytoscape 3.6.1. Except AtRFL10, which did not have interaction information in the STRING database, 25 AtRFLs and their predicted partners are shown in Fig. 4 and Additional file 4. Interestingly, AtRFL11, AtRFL12 and AtRFL13 and three HXKs, including HXK1, HXK2 and HXK3, shared a common interacting protein, namely replication factor C2 (RFC2), a multi-subunit complex critical for high-speed ATP-dependent DNA synthesis [33]. No homologs of *AtRFL25* were identified in *B. napus*. Approximately one-quarter of the *BnRFL* genes were homologous to *AtRFL2* and *AtRFL3*. Further analysis revealed that AtRFL2 and AtRFL3 interact with AtRFL25, which showed interaction with the glycine-rich proteins, GRP7 and GR-RBP2 (Fig. 4). Moreover, AT1G48510, SURF1, COX15 and COX11 were predicted to interact with atp6–1, AT3G48810, NAD9, CCMH and most of the AtRFLs.

### Expression analysis of *BnRFL* genes

**Fig. 5:**
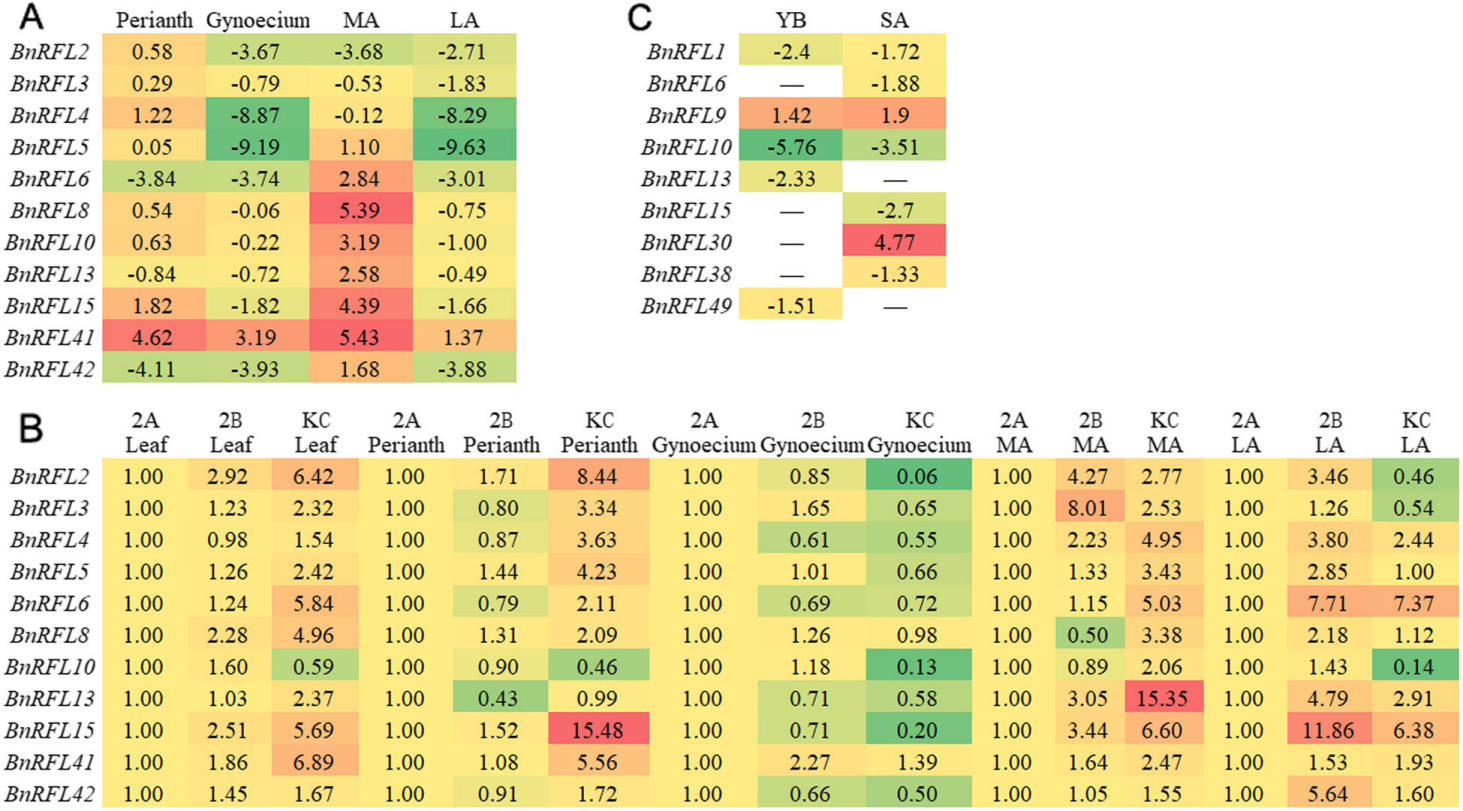
Expression profiles of *BnRFL* genes. **a** Expression profiles of *BnRFL* genes in different tissues of KC01. Gene expression in other tissues was calculated relative to that in leaves. The log_2_^X^-normalized ratios are shown. **b** Comparative expression analysis of *BnRFL* genes in different tissues of KC01, Shaan2B and Shaan2A. Gene expression levels in KC01 and Shaan2B was calculated relative to those in Shaan2A. **c** RNA-seq analysis of the differentially expressed *BnRFLs* at YB and SA stage. Gene expression levels in Shaan2A was calculated relative to those in KC01. Red indicated higher expression levels. Green represented the lower expression levels. ‘-’ indicates no significant difference in expression

Based on the results of phylogenetic analysis, 12 *BnRFL* genes, which were mentioned in the phylogenetic analysis, were selected for tissue-specific expression analysis in the sterile line Shaan2A, the maintainer line Shaan2B and the restorer line KC01 by qRT-PCR. Although *BnRFL10* and *BnRFL11* were located on different chromosomes, the coding sequence (CDSs) of these genes were highly similar (identity = 1926/1947; 99%), and it was difficult to distinguish them by qRT-PCR. Therefore, we finally analyzed the expression of 11 *BnRFL* genes. In the restorer line, the expression of *BnRFL6*, *BnRFL13* and *BnRFL42* was lower in leaves than in the perianth (Fig. 5a, Additional file 5). The majority of the selected *BnRFLs* showed higher expression level in MA when compared with leaves, except for *BnRFL2*, *BnRFL3* and *BnRFL4*. However, the expression of the *BnRFL* genes, except *BnRFL41*, was lower in the gynoecium and LA when compared with leaves. Compared with Shaan2A tissues, the expression of 11 *BnRFL* genes was higher in KC01 tissues, especially in MA (Fig. 5b, Additional file 5). What’s more, the expression of these genes in Shaan2B LA was higher than those in Shaan2A (Fig. 5b, Additional file 5). However, the expression of most of these *BnRFLs* was lower in the gynoecium of the restorer line than in that of Shaan2A.

To compare the expression level of all 53 *BnRFL* genes in Shaan2A vs. KC01, three biological replicates of RNA-seq were performed using RNA isolated from young buds (YB, < 1 mm, representing pre-meiosis) and small anthers (SA, sampled from buds 1–2 mm in length (representing tetrad stage to microspore release stage). A total of 320,892,232 raw sequence reads were generated, with approximately 50 million raw reads representing each tissue sample (SRA number: PRJNA511929). Additionally, to conduct comparative transcriptome analysis of the three lines in the Shaan2A CMS system, raw transcriptome reads representing the same stages of Shaan2A and Shaan2B were downloaded (SRA number: PRJNA502996). RNA-seq data analysis of Shaan2A and KC01 revealed only nine *BnRFLs* exhibited differential expression (Fig. 5c). These results provide important clues for analyzing the candidate restorer genes in the Shaan2A CMS system.

### Transcriptomic analysis between Shaan2A and KC01

To investigate the differences between Shaan2A and KC01, possibly caused by the male sterile genes and restorer genes, we also identified the differentially expressed genes (DEGs) between Shaan2B and KC01, as Shaan2A and Shaan2B share the same nuclear genetic background. Thus, common DEGs identified based on Shaan2A vs. KC01 comparison and Shaan2B vs. KC01 comparison would represent the DEGs identified between different genetic backgrounds, i.e., Shaan2A (or Shaan2B) and KC01. A total of 2980 and 8243 DEGs were identified in YB and SA stage, respectively, based on the comparison between Shaan2A and KC01 (|log2 Ratio| > 1; Additional file 6).

**Fig. 6:**
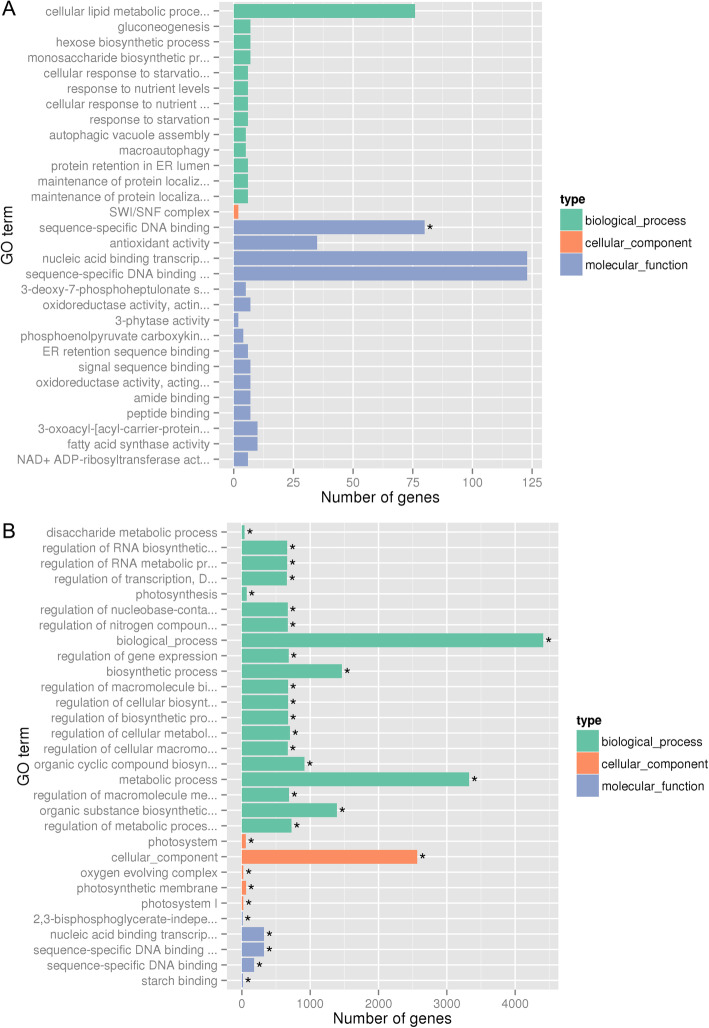
Top 30 gene ontology (GO) sub-categories of the DEGs identified between Shaan2A and KC01. Asterisk indicates the corrected *p*-value < 0.05. **a** YB stage. **b** SA stage

Based on GO analysis, only one GO term in the molecular function category, ‘sequence-specific DNA binding’, was significant at YB stage (Fig. 6a). By contrast, at SA stage, 8243 DEGs identified between Shaan2A and KC01 were categorized in three main categories, including molecular function, cellular component and biological process, which were further classified into many functional sub-categories (Additional file 7). The top 30 sub-categories, including ‘disaccharide metabolic process’, ‘regulation of RNA biosynthetic process’, ‘regulation of RNA metabolic process’ and ‘regulation of transcription, DNA-dependent’, are shown in Fig. 6b.

To validate the RNA-seq data, the expression of 11 DEGs, potentially involved in anther development, was analyzed by qRT-PCR (Additional file 8). The results of qRT-PCR analysis for most of these DEGs at the two stages were consistent with those of RNA-seq analysis, indicating that the reliability of our RNA-seq data.

## Discussion

Many CMS systems have been used in *B. napus*, including *pol*, *nap*, *Ogu* and Shaan2A [4–7]. To date, *Rf* genes have been identified via genetic mapping only in *pol* and *nap* CMS systems [17, 18]. In the present study, 53 *BnRFL* genes were identified in the Shaan 2A CMS system. Most of these genes contained more than 10 PPR motifs, which is consistent with the previously reported restorer proteins such as Rf-PPR592 (14 PPR motifs), Rf4 (18 PPR motifs) and Rfo (17 PPR motifs) [11, 17, 24]. Moreover, most of the BnRFLs identified in this study were predicted to localize to mitochondria, similar to the known restorer genes [15–18]. Since the *Rf* genes function with the toxic chimeric genes in mitochondria to rescue male sterility [14–16], the mitochondrial localization of the proteins seems appropriate. More importantly, we also identified *BnRFL6* (*Rfn*) and *BnRFL13* (*Rfp*), previously confirmed as restorer genes in *nap* and *pol* CMS systems, respectively [17, 18], and four candidate restorer genes (*BnRFL2*, *BnRFL10*, *BnRFL11* and *BnRFL42*), previously identified in *B. napus* via genetic mapping [31]. Taken together, analysis of the *RFL* gene family for the identification of candidate restorer genes were viable, which would also provide a new way to analysis the restorer genes in other CMS systems as one supplementary method except for the traditional genetic mapping to locate the candidate genes.

Nearly 7500 years ago, *B. napus* originated from the hybridization of *B. rapa* and *B. oleracea* [34], and the Brassica plants experienced the extra whole genome triplication (WGT) event when compared with Arabidopsis [35]. The *Arabidopsis* genome contains 26 *RFL* gene family members, so considering the WGT event there should be over 78 *RFL* genes in *B. oleracea* or *B. rapa* genome, and finally generate even more *RFL* genes in *B. napus*. While only 53 *BnRFL*s were identified in the present study, which implied that nearly 50% *RFL* genes were lost after the WGT event.

Most of the *BnRFLs* were unevenly distributed on 10 of the 19 chromosomes of *B. napus*, while a few formed gene clusters on chromosomes A9 and C8, similar to the gene cluster in *Arabidopsis* (chromosome 1; Table 1, Fig. 1), rice and barley (*Hordeum vulgare*) [36, 37]. Gene clustering has also been observed in many other gene families, such as the *LEA* gene family in *B. napus* [38] and *Phyllostachys edulis* [39] and laccase gene family in *Citrus sinensis* [40]. The *LEA* gene clusters on *B. napus* chromosomes A9 and C4 probably resulted from chromosomal rearrangement during the evolution of *Brassica* species [38]. The *RFL* genes on *Arabidopsis* chromosome 1 showed synteny with the *RFL* genes on BraA9, BolC8, BnaA9 and BnaC8. Additionally, *BnRFLs* maintained a syntenic relationship with *RFL* genes in *B. rapa* and *B. oleracea*, suggesting that a conserved role of *BnRFLs* located on chromosomes A9 and C8. Moreover, *AtRFL2*, *AtRFL4* and *AtRFL9* were located within the gene cluster on *Arabidopsis* chromosome 1 and participated in the processing of the mitochondrial RNA [25–27]. The phylogenetic analysis revealed that the BnRFLs have the closer phylogenetic relationship with AtRFLs and RsRFLs (Fig. 3), and the structural analysis showed that all of the BnRFLs and the known restorer genes in radish share a conserved motif (Fig. 2), and all *BnRFL* genes showed a Ka/Ks ratio < 1 (Additional file 3), which indicated that there was no positive selection on the *BnRFL* genes during the evolution. What’s more, *BnRFL6* (*Rfn*) and *BnRFL13* (*Rfp*) were located within the gene cluster on chromosome A9. These data suggest that the *RFL* genes within gene clusters on chromosomes A9 and C8 represent the restorer genes in the CMS system, as these likely exhibit a conserved role in mitochondrial RNA processing.

Tandem repeats of a degenerate 35-amino-acid PPR motif are the most prominent feature of the PPR family, and all of the 53 BnRFL proteins showed this trait. Although 212 *RFL* genes in 13 different species [24] and 26 *RFL* genes in barley [37] have been identified previously, the conserved domain of the RFL proteins has not yet been analyzed. Therefore, we investigated motifs other than PPR in the RFL proteins, revealing 20 motifs among the 53 BnRFLs and a few known RF proteins. Interestingly, all RFLs contained motif 1, comprising 80 amino acids. We propose motif 1 as the conserved motif in the RFL protein family. This motif will serve as a reference for RFL family analysis in other species.

Because RF-related PPR proteins belong to the P subfamily and do not exhibit endonuclease activity, these proteins form functional complexes with other proteins [20]. To date, only two RFL-interacting partner proteins have been identified, including GRP162 and HXK6 in the rice CMS system [15, 16]. In the present study, we constructed an interaction network for AtRFLs (Fig. 4). Interestingly, AtRFL11, AtRFL12 and AtRFL13 and HXKs (HXK1, HXK2 and HXK3) shared a common interacting partner, RFC2, which was critical for high-speed DNA synthesis [22], whereas AtRFL25 showed interaction with GRP7 and GR-RBP2. Moreover, AT1G48510, SURF1, COX11 and COX15 were predicted to interact with most of the AtRFLs. AT1G48510 is a surfeit locus 1 cytochrome c oxidase biogenesis protein. SURF1 is associated with cytochrome c oxidase assembly [41]. Both COX11 and COX15 are mitochondrial proteins and belong to the cytochrome c oxidase protein family [42]. COX11 likely plays a key role as a mitochondrial chaperone in the assembly of the COX complex and regulates pollen germination and plant growth [43]. Overall, the interaction network indicates possible partner proteins of RFL proteins in *Arabidopsis*. These data provide important clues for the identification of interaction factors of RF proteins in other species.

Previously, it has been shown that *Rf4* is constitutively expressed in different rice organs at relatively low levels [14]. Although *Rf6* expression is detectable in various rice tissues, it is expressed to a higher level in the panicle than in other tissues [16]. In the *pol* CMS system, *Rfp* shows relatively high expression in flower buds and weak expression in opening flowers, leaves, stems and roots [17]. In the phylogenetic tree, two previously reported restorer genes (*BnRFL6* and *BnRFL13*), four candidate restorer genes (*BnRFL2*, *BnRFL10*, *BnRFL11* and *BnRFL42*) and another six *BnRFLs* (*BnRFL3*, *BnRFL4*, *BnRFL5*, *BnRFL8*, *BnRFL15* and *BnRFL41*) clustered together, suggesting these genes as the more probable candidates of restorer genes in the rapeseed CMS system. Analysis of expression patterns revealed that most of these genes, except for *BnRFL2*, *BnRFL3* and *BnRFL4*, were expressed to relatively higher levels in MA than in leaves in KC01. Additionally, these *BnRFLs* showed higher expression in KC01 tissues, especially MA, than in Shaan2A tissues. Expression profiling of *BnRFL* genes in Shaan2A vs. KC01 showed that *BnRFL1*, *BnRFL6*, *BnRFL10*, *BnRFL13*, *BnRFL15*, *BnRFL38* and *BnRFL49* were down-regulated in Shaan2A. However, BnRFL15 only harbored five PPR motifs, which was much lower than the number of PPR motifs in the known RF proteins. While *BnRFL38* and *BnRFL49* are located on chromosomes A1 and A10, respectively, *BnRFL1*, *BnRFL5* and *BnRFL8*, *BnRFL6*, *BnRFL11*, *BnRFL13* and *BnRFL42* are located in gene clusters on chromosomes C8 and A9. Interestingly, almost all of the known rice *Rf* genes are located in the *RFL* gene cluster on chromosome 10 [36]. These data suggest *BnRFL1*, *BnRFL5*, *BnRFL6*, *BnRFL8*, *BnRFL11*, *BnRFL13* and *BnRFL42* as the more likely candidates of restorer genes in the Shaan2A CMS system. In *O. sativa*, RF6 with a characteristic duplication of PPR motifs in the restorer line of Honglian CMS can restore sterility, while the duplicated motifs are absent in rf6 of sterile line [16]. In the next steps, we will clone these candidate restorer genes in the restorer line and sterile line of Shaan2A CMS respectively, and compare the difference of sequences between them, for we wonder if there is the similar motif difference between these candidate restorer genes. Then we will narrow down the list of candidate genes, and conduct the transgenic work in sterile line to investigate their function.

Furthermore, DEGs identified in small anthers of Shaan2A vs. KC01 were annotated as involved in the ‘regulation of RNA biosynthetic process’, ‘regulation of RNA metabolic process’ and ‘regulation of transcription, DNA-dependent’. The RF-related PPR proteins interact with their partner proteins to bind or to edit RNA [15]. Here, the regulation of RNA biosynthetic, RNA metabolic process and transcription was different between the sterile line and restorer line, which might be caused by the sterile genes in Shaan2A and restorer genes in KC01. However, the detailed mechanism needs further investigation.

## Conclusions

In CMS, the *Rf* nuclear genes rescue the sterile phenotype and most of the *Rf* genes encode pentatricopeptide repeat (PPR) proteins. In the present study, a total of 53 *BnRFL* genes were identified in *B. napus*. Most of the *BnRFL* genes were distributed on 10 of the 19 chromosomes, and gene clusters were identified on chromosomes A9 and C8. The interaction network analysis was performed to predict the interacting partners of RFL proteins. Tissue-specific expression and RNA-seq analyses between the restorer line KC01 and the sterile line Shaan2A indicated that *BnRFL1*, *BnRFL5*, *BnRFL6*, *BnRFL8*, *BnRFL11*, *BnRFL13* and *BnRFL42* located in gene clusters on chromosomes A9 and C8 were highly expressed in KC01, which suggest these seven *BnRFL* genes as strong candidates for the restorer genes in Shaan2A CMS. Our results would provide new insight into the study of *Rf* genes in other CMS systems.

## Methods

### Plant materials

The sterile line Shaan2A, maintainer line Shaan2B and restorer line KC01 of *B. napus*, gifted by Professor Dianrong Li at the Hybrid Rape Research Center of Shaanxi Province, were used in this study. Plants were cultivated on the experimental field of the Huazhong University of Science and Technology (Wuhan, Hubei province, China). After harvest, plant samples were immediately frozen in liquid nitrogen and stored at − 80 °C until needed for total RNA isolation.

### Identification of the RFL gene family in *B. napus* and other species

The *RFL* genes were identified as described previously [24]. Briefly, AtRFL1–26 [24] and Rf-PPR592 [11] sequences were used for BLAST searches against the genome of the rapeseed cultivar ‘ZS11’ [44]. The sequence of rice RF5 (also known as RF1a) [15, 45] was used for BLAST searches against the genome sequences of monocots (E-value <1e^− 100^), including *O. sativa* (RGAP, http://rice.plantbiology.msu.edu/) and *Z. mays* [46]. The Rf-PPR592 sequence was used for BLAST searches against the genome sequences of dicots (E-value <1e^− 100^), including *B. rapa* [47], *B. oleracea* [48] and *R. sativus* [49].

The grand average of hydropathy (GRAVY) value, isoelectric point (pI) and molecular weight of RFL proteins were calculated using ExPASy (http://www.expasy.org/tools/) [28]. The subcellular location of RFL proteins was predicted using the Protein Prowler Subcellular Localization Predictor version 1.2 (http://bioinf.scmb.uq.edu.au:8080/pprowler_webapp_1-2/) [29] and TargetP1.1 server (http://www.cbs.dtu.dk/services/TargetP/) [30].

### Structural analysis of RFL genes

The exon-intron structure of *BnRFL* genes and a few known *Rf* genes were based on the alignments of the CDS with the corresponding genomic sequences, and the diagram was conducted using the Gene structure display server (GSDS, http://gsds.cbi.pku.edu.cn/) [50]. The PPR motifs in all BnRFL proteins and a few known RF proteins were analyzed using the NCBI Conserved Domain Search tool (http://www.ncbi.nlm.nih.gov/Structure/cdd/wrpsb.cgi) [51]. Conserved motifs in RFL proteins were analyzed using Multiple Expectation Maximization for Motif Elicitation (MEME, http://alternate.meme-suite.org/) [52].

### Phylogenetic and Syntenic analysis of RFL genes

Multiple sequence alignment of the predicted amino acid sequences of BnRFLs, AtRFLs, RsRFLs, OsRFLs and ZmRFLs was performed using ClustalX [53]. A phylogenetic tree of these RFL proteins was constructed with MEGA 7 using the Neighbor Joining (NJ) method [54]. Analysis of synteny among *BnRFL*, *AtRFL*, *BoRFL* and *BrRFL* genes was performed using the syntenic gene tool in the Brassica database (BRAD, http://brassicadb.org/brad/) [55]. The non-synonymous to synonymous nucleotide substitution ratio (Ka/Ks) was calculated using TBtools [56].

### Interaction analysis

The interaction analysis of AtRFLs was based on the STRING 10.5 database, which included the known and predicted protein–protein interactions. First, the interaction proteins of AtRFLs were searched. After deleting the repeat proteins, the interaction network was visualized using Cytoscape 3.6.1.

### RNA extraction, RNA-seq and qRT-PCR analysis

Gene expression was analyzed in various tissues of the sterile line Shaan2A and restorer line KC01 including leaves, perianths, gynoecium, medium anthers (MA) and large anthers (LA). MA were harvested from buds 2–4.5 mm in length and represented the uninuclear microspore stage, whereas LA were harvested from buds 4.5 mm in length and represented the mature pollen formation stage.

Total RNA extraction, RNA-seq analysis and qRT-PCR were conducted according to our previous protocols [57], with minor modifications. Briefly, approximately 100 mg plant samples were used for total RNA extraction using TRIzol Reagent (Invitrogen, Carlsbad, CA, USA), according to the manufacturer’s instructions. Then, cDNA sequencing libraries were constructed using TruSeq™ RNA Sample Preparation Kit (Illumina, San Diego, CA, USA). RNA-seq was performed on the Illumina NovaSeq 6000 platform. The raw data were filtered using the NGSQC toolkit (v2.2.3), and the clean reads were mapped to the reference genome of the rapeseed cultivar ‘ZS11’. The differentially expressed genes (DEGs) were evaluated using DESeq2, with normalized fold-change ≥2 and *p*-value < 0.05. Gene Ontology (GO) annotation was using the Web Gene Ontology Annotation Plot (WEGO) software.

To perform qRT-PCR analysis, RNA was reverse transcribed using the TaKaRa PrimeScript™ RT Reagent Kit with gDNA Eraser, according to the manufacturer’s instructions. *Actin* was used as the internal reference gene [58]. The qRT-PCR experiments and transcript quantification were performed as described previously [57]. Primers used in this study are listed in Additional file 9.

## Supplementary Information

**Additional file 1 **Exon-intron structure of the *BnRFL* genes and known *Rf* genes.

**Additional file 2.** Distribution of PPR motifs in the identified RFL proteins.

**Additional file 3 **Non-synonymous (Ka) and synonymous (Ks) nucleotide substitution rates of the coding sequence of *RFL* genes in *A. thaliana* and *B. napus*.

**Additional file 4.** List of the interaction proteins.

**Additional file 5.** Original data of qRT-PCR.

**Additional file 6.** RNA-seq analysis of DEGs identified between Shaan2A and KC01 at YB and SA stage, respectively.

**Additional file 7.** List of GO sub-categories of the DEGs identified between Shaan2A and KC01.

**Additional file 8.** Validation of the expression of selected DEGs by qRT-PCR. (A) Results of qRT-PCR analysis. (B) Results of RNA-seq analysis. The numbers indicate log_2_^X^-normalized ratios. Red indicated higher expression levels. Green represented the lower expression levels. ‘-’ indicates no significant difference in RNA-seq data.

**Additional file 9.** List of primers used in this study.

## Data Availability

Raw RNA-seq data of KC01 were submitted to the NCBI Sequence Read Archive (SRA) database under the accession number PRJNA511929. Raw RNA-Seq data of Shaan2A and Shaan2B (accession no. PRJNA502996) were downloaded from the NCBI SRA database [59]. The reference genome of the rapeseed cultivar ‘ZS11’, *B. rapa*, *B. oleracea*, *R. sativus* and *Z. mays* are available at NCBI under the project ID of PRJNA394926, PRJNA249065, PRJNA59981, PRJNA293438, PRJNA344915, PRJNA655717 and PRJEB32225 respectively [41, 43–46]. The reference genome of *O. sativa* was available at Rice Genome Annotation Project (RGAP, http://rice.plantbiology.msu.edu/). *BnRFL1–53* can be found with NCBI accession numbers as LOC106420094, LOC106397711, LOC106397817, LOC106412080, LOC106412541, LOC106397421, LOC106350729, LOC111208528, LOC106382383, LOC106380919, LOC106369154, LOC106362038, LOC106401178, LOC106359321, LOC111208839, LOC106412542, LOC106400043, LOC106436889, LOC106368851, LOC106348977, LOC106367812, LOC106368854, LOC106395610, LOC106362947, LOC106377687, LOC106448592, LOC106448594, LOC106373934, LOC106360986, LOC106450684, LOC106450694, LOC106390267, LOC106366458, LOC106358569, LOC106420242, LOC106450895, LOC111211867, LOC106390802, LOC106437800, LOC106382376, LOC106416119, LOC106397756, LOC106423886, LOC106378791, LOC111208626, LOC106444978, LOC106445419, LOC106432155, LOC106371992, LOC106435274, LOC106446207, LOC106367284, LOC106411529 respectively. The GRAVY value, pI and molecular weight of RFL proteins were calculated using ExPASy (http://www.expasy.org/tools/) [28]. The subcellular location of RFL proteins was predicted using the Protein Prowler Subcellular Localization Predictor version 1.2 (http://bioinf.scmb.uq.edu.au:8080/pprowler_webapp_1-2/) [29] and TargetP1.1 server (http://www.cbs.dtu.dk/services/TargetP/) [30] respectively.

## Abbreviations

*RFL*: *Restorer-of-fertility-like*
CMS: Cytoplasmic male sterility
*Rf*: *Restorer-of-fertility*
PPR: Pentatricopeptide repeat
CIMS: Chemical induced male sterility
GMS: Genic male sterility
HXK6: Hexokinase 6
RPF4: RNA PROCESSING FACTOR 4
TAIR: The Arabidopsis Information Resource
RFC2: Replication factor C2
GRAVY: Grand average of hydropathy
CDS: Coding sequence
GSDS: Gene structure display server
NJ: Neighbor Joining
DEG: Differentially expressed gene
GO: Gene Ontology
