# Supplementary figures and images for: Genome-wide identification of the restorer-of-fertility-like (RFL) gene family in Brassica napus and expression analysis in Shaan2A cytoplasmic male sterility
Source: BMC Genomics. 2020 Nov 4;21:765. doi: 10.1186/s12864-020-07163-z (PMC7641866; doi:10.1186/s12864-020-07163-z)

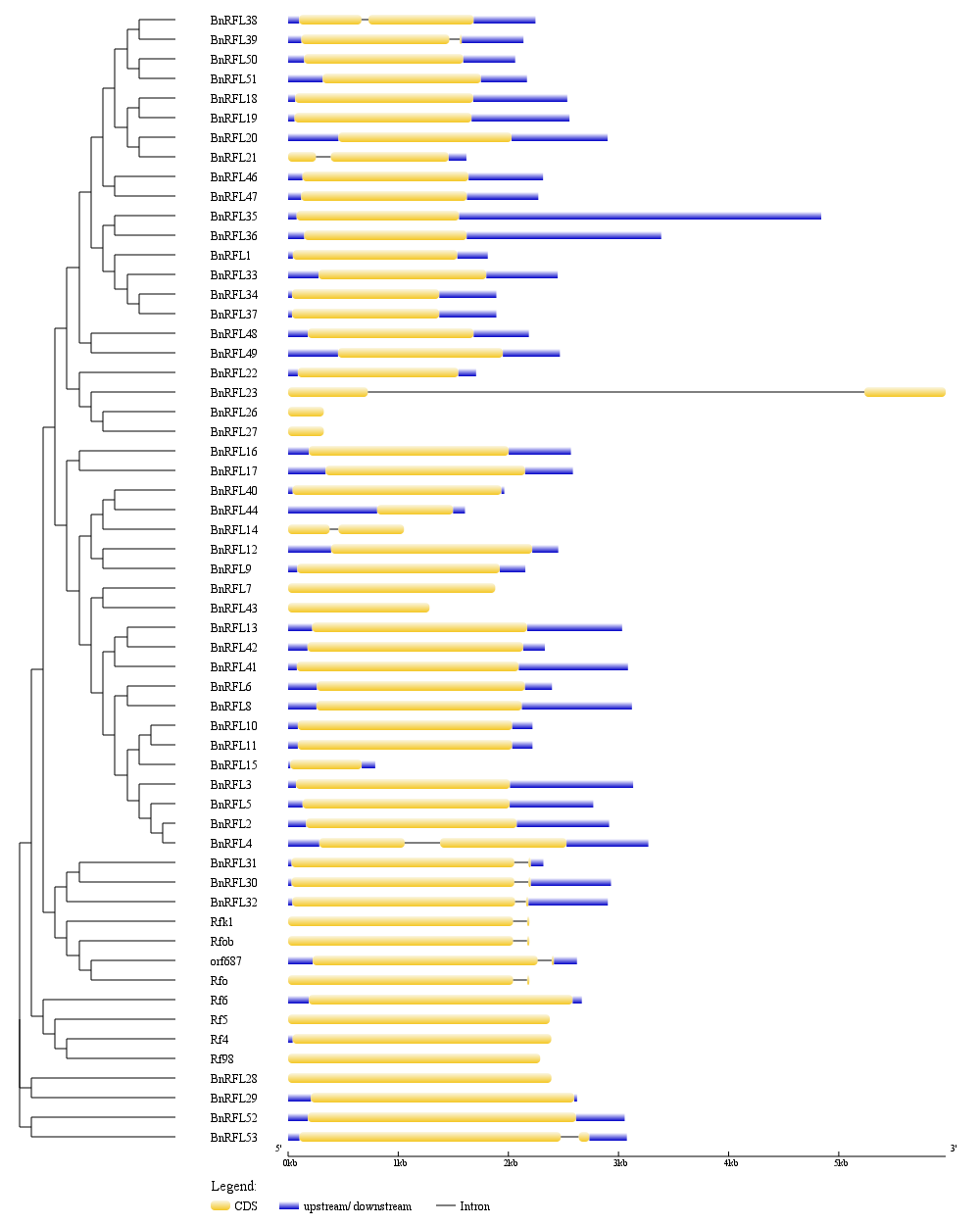

Supplement: Supplementary file 1 — Additional file 1 Exon-intron structure of the BnRFL genes and known Rf genes. [file 12864_2020_7163_MOESM1_ESM.png]

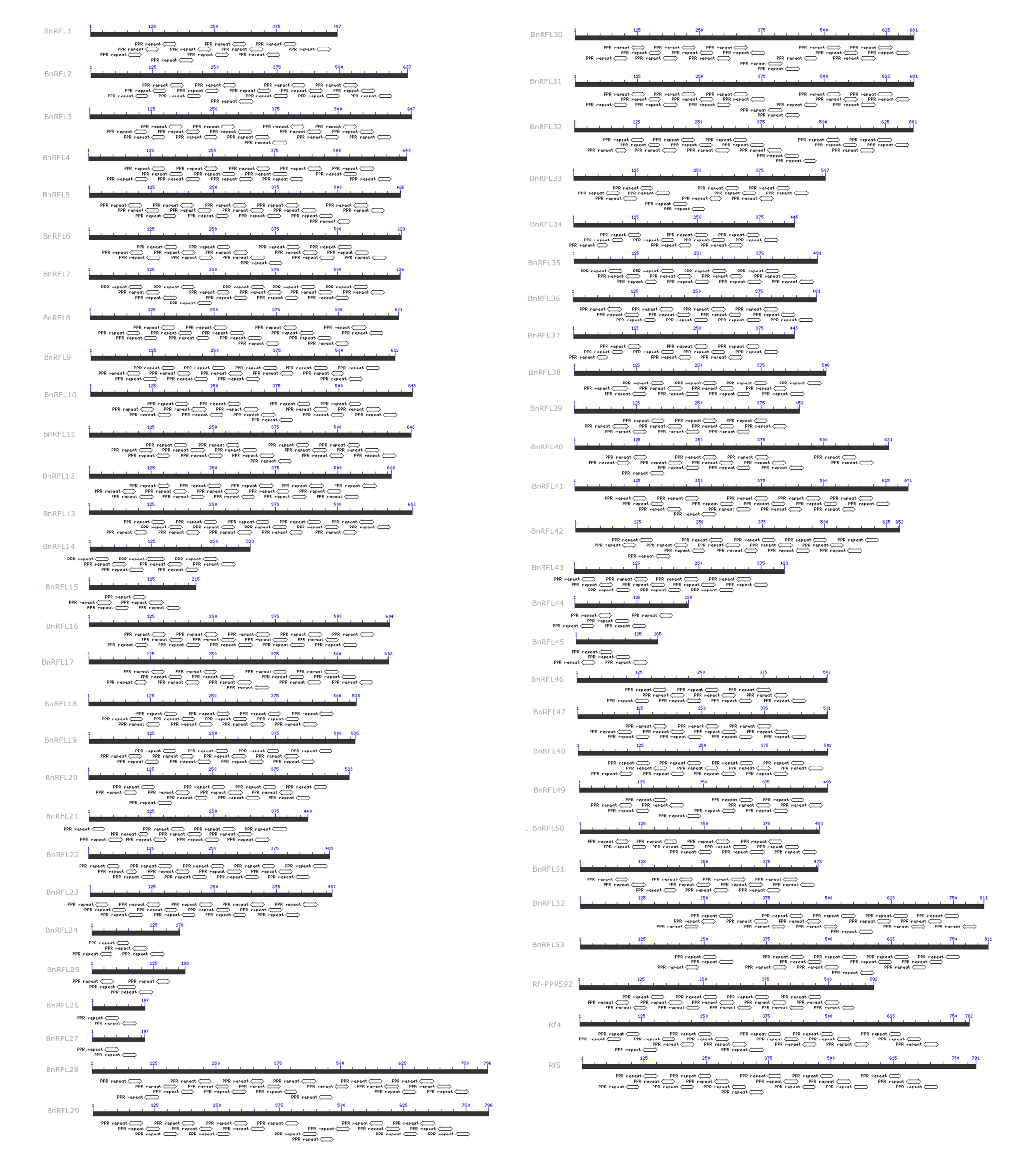

Supplement: Supplementary file 2 — Additional file 2. Distribution of PPR motifs in the identified RFL proteins. [file 12864_2020_7163_MOESM2_ESM.jpg]

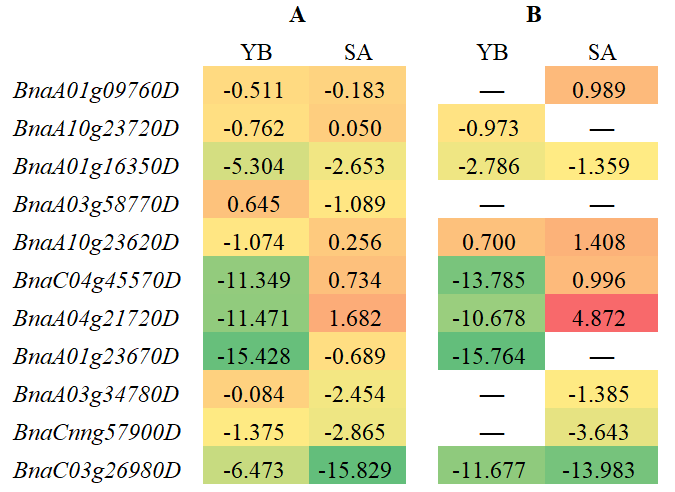

Supplement: Supplementary file 8 — Additional file 8. Validation of the expression of selected DEGs by qRT-PCR. (A) Results of qRT-PCR analysis. (B) Results of RNA-seq analysis. The numbers indicate log2X-normalized ratios. Red indicated higher expression levels. Green represented the lower expression levels. ‘-’ indicates no significant difference in RNA-seq data. [file 12864_2020_7163_MOESM8_ESM.png]
